# Principles of Medicine, Comprising General Pathology and Therapeutics, and a Brief General View of Etiology, Nosology, Semeiology, Diagnosis, Prognosis and Hygienics

**Published:** 1848-08

**Authors:** 


### BIBLIOGRAPHICAL NOTICES.

*Principles of Medicine, comprising General Pathology and Therapeutics, and a brief general view of Etiology, Nosology, Semeiology, Diagnosis, Prognosis and Hygienics.* By CHARLES J. B. WILLIAMS, M. D., F. R. S., Fellow of the Royal College of Physicians, &c., &c. *Edited, with additions, by* MEREDITH CLYMER, M. D., Fellow of the Philadelphia College of Physicians, late Professor of the Principles and Practice of Medicine, and of Clinical Medicine, in the Franklin Medical College: Consulting Physician to the Philadelphia Hospital, &c., &c. *Third American from the Second and enlarged London edition.* 8vo. pp. 440. Philadelphia. Lea & Blanchard: 1848.

We have before directed attention to the valuable work of Dr. Williams with the appropriate additions to it by the capable American editor. If it were then worthy of commendation, it is still more so now. It is a work of thought,—of thought suggestive of observation, and of thought suggested by observation; and is, consequently, eminently progressive in its tendency and scope. "It possesses," the American editor remarks, "the strongest claims to the attention of the medical student and practitioner, from the admirable manner in which the various enquiries in the different branches of pathology are investigated, combined, and generalized by an experienced practical physician, and directly applied to the investigation and treatment of disease."

An editor may naturally be supposed to be biassed in favour of the work to which he is sponsor and prolegomenon. No one, however, is better qualified to appreciate such a production than he who acts as such on the present occasion. As Professor of the "Principles" of Medicine in the Franklin Medical College, the whole subject must have fallen under his serious attention, had not his studies previously led him in that direction. We know, however, that at all times the investigation of the phenomena of disease, and of the laws of those phenomena, has been closely and successfully pursued by him; and hence no one could have been selected with more—and few with as much—propriety to edit a work like the one before us. Of the nature and character of this work we shall not repeat what was said by us of the former editions. The author affirms that he has made "very extensive additions" to the present issue, comprising the enumeration and application of most of the facts and established deductions made available to the science and art of medicine during the last few years. These additions pervade almost every portion of the work; but they preponderate in the following subjects:

"In Etiology, *mechanical, chemical*, and dietetic *causes of disease, defective cleanliness, ventilation*, and *drainage*. In Pathology, the tabular views of the elements of disease; *reflex action and symputhy; elementary changes in the blood; congestion; determination of blood; inflammation* in its nature, manifold results, and modes of treatment; *degeneration of textures; cacoplastic and aplastic deposits*, and their treatment, with a notice of the action of *cod liver oil;* and the whole chapter on *Hygienics*, comprising *food, clothing, air*, and *temperature, exercise, mental occupation, sleep* and *excretion.* p. vii.

"The chief additions by the editor will be found in the sections on Etiology, Diseases of the Constituents of the Blood, Nutrition, Semeiology, Prognosis, and Hygienics. The section on Semeiology has been considerably enlarged, and an abstract of the general signs of disease added."

We once more, and not—we trust—for the last time, commend this work to the attention of our readers. It is one of the few productions to which they can refer with satisfaction for the expositions of a sound and rational pathology.

